# Limb Salvage After Failed Infected Total Knee Arthroplasty: A Comparison of Cement-Based Artificial Arthrodesis and External Fixator Arthrodesis

**DOI:** 10.3390/life16101721

**Published:** 2026-10-15

**Authors:** Savaş Çamur, Murat Birinci, Serkan Bayram, Bahattin Kemah, Muhammet İbrahim Karaçam, Ömer Polat, Çağrı Özcan, Halil İbrahim Balcı

**Affiliations:** 1 Department of Orthopaedics and Traumatology, Umraniye Training and Research Hospital, 34764 İstanbul, Türkiye; drmuratbirinci@gmail.com (M.B.);; 2 Department of Orthopaedics and Traumatology, Faculty of Medicine, İstanbul University, 34093 İstanbul, Türkiye

**Keywords:** knee fusion, periprosthetic joint infection, antibiotic-loaded cement, functional outcomes, limb length discrepancy

## Abstract

**Background:** Persistent or recurrent periprosthetic joint infection after total knee arthroplasty is a devastating complication. When revision fails, knee arthrodesis remains an important limb-salvage option. Cement-based artificial arthrodesis may provide an alternative when extensive bone loss or soft-tissue compromise limits conventional fusion, but comparative evidence is scarce. **Methods:** This retrospective exploratory cohort included 36 patients whose index salvage procedures were performed between January 2014 and December 2023 after failed treatment of periprosthetic knee infection. Sixteen patients underwent external fixator arthrodesis, and 20 underwent cement-based artificial arthrodesis. Postoperative SF-12 and SF-36 scores, ambulation, limb shortening, extensor mechanism status, microbiological findings, reinfection, and complications requiring surgery were evaluated. **Results:** The groups differed at baseline: preoperative ESR and CRP were higher in the cement-based group, whereas extensor mechanism insufficiency was more frequent in the external fixator group (81.3% vs. 20%; *p* = 0.001). No statistically significant between-group differences were detected in most SF-12 and SF-36 domains; Energy/Fatigue was higher in the external fixator group (*p* = 0.011), an isolated exploratory finding. Mean postoperative shortening was numerically greater after external fixation (4.94 ± 3.66 vs. 3.15 ± 0.49 cm; *p* = 0.07). Complications requiring surgical intervention occurred in 31.2% and 25% of patients, respectively. Reinfection requiring additional surgery occurred in 0/16 external fixator patients and 1/20 cement-based patients. **Conclusions:** Cement-based artificial arthrodesis may represent a practical limb salvage option in selected patients with failed infected TKA, particularly when direct bone apposition is difficult. Because treatment allocation was non-random, baseline characteristics were imbalanced, and the cohort was small, so the findings should be interpreted as exploratory and not as evidence of equivalence or superiority.

## 1. Introduction

Periprosthetic joint infection (PJI) remains one of the most devastating complications following total knee arthroplasty (TKA). Contemporary studies report an incidence of approximately 0.5–2% after primary TKA, although estimates vary according to case definition, follow-up, and patient population [1,2]. PJI is also a major cause of failure after knee arthroplasty and is associated with repeated operations, prolonged antimicrobial treatment, and substantial healthcare utilization [3]. Recent cost analyses further demonstrate the considerable economic burden associated with treatment of hip and knee PJI [4].

Chronic or recurrent PJI is generally managed with one-stage or two-stage revision; however, infection eradication may not be achieved in a subset of patients, particularly after repeated procedures or in the presence of extensive bone loss, soft-tissue compromise, resistant or polymicrobial infection, and poor host status [5,6]. When further revision arthroplasty is unlikely to provide a durable reconstruction, salvage options include above-knee amputation and knee arthrodesis [7,8]. Knee arthrodesis can provide limb stability and preservation of ambulation in complex failures of TKA [9].

Knee arthrodesis may be performed using external fixation, intramedullary fixation, or plate fixation. External fixation remains particularly relevant in the setting of infection because it avoids permanent hardware within the infected medullary canal and permits compression and adjustment of alignment during fusion [10,11]. Nevertheless, prolonged frame use may be associated with pin-site complications, patient discomfort, rehabilitation challenges, and the need for adequate bone contact to obtain osseous fusion [11]. A recent systematic review comparing arthrodesis techniques for PJI found broadly similar fusion and reinfection outcomes between external fixation and intramedullary fixation [12].

However, severe metaphyseal bone loss after repeated debridement may preclude conventional bone-to-bone fusion or require substantial limb shortening. Sarzaeem et al. described a modified artificial fusion technique using cement-filled intramedullary rods in unreconstructible revision TKA [13], while DeBaun et al. reported cement arthrodesis using a long reconstruction nail after failed TKA [14]. Permanent or retained cement-based constructs have also been reported as definitive treatment in selected high-risk patients [15,16]. Despite these reports, the available evidence remains dominated by small case series, and direct comparative data between cement-based artificial arthrodesis and external fixator arthrodesis are scarce.

Accordingly, the objective of this study was to compare the observed functional outcomes, postoperative limb shortening, microbiological characteristics, and complications of external fixator arthrodesis and cement-based artificial arthrodesis performed as salvage procedures for persistent or recurrent PJI following TKA. Given the retrospective design and non-random treatment allocation, the study was intended as an exploratory comparison of two limb salvage strategies rather than an assessment of treatment superiority or equivalence.

## 2. Materials and Methods

### 2.1. Patients and Study Design

This retrospective comparative cohort study was conducted at two tertiary orthopedic centers and included patients with persistent or recurrent PJI following TKA who underwent the index salvage procedure between January 2014 and December 2023. The study was approved by the institutional ethics committee and was conducted in accordance with the Declaration of Helsinki.

Adult patients (≥18 years) who developed PJI after primary or revision TKA and subsequently underwent definitive limb salvage treatment with either external fixator arthrodesis or cement-based artificial arthrodesis were considered eligible. The diagnosis of PJI was established according to the 2018 Musculoskeletal Infection Society (MSIS) criteria [17]. For the purposes of this study, the index salvage procedure was defined as the definitive arthrodesis procedure analyzed in the present cohort. Patients could have undergone previous debridement procedures, static or dynamic antibiotic spacer placement, failed two-stage revision arthroplasty, or other infection-related surgical interventions before the index salvage procedure; however, none had undergone a previous knee arthrodesis. A minimum postoperative follow-up of 12 months was required.

The choice of salvage procedure was not based on a predefined treatment algorithm. Treatment selection was primarily determined by the treating surgeon’s preference together with the patient’s anatomical and clinical characteristics. Accordingly, treatment allocation was non-random and may have been influenced by factors including bone loss, soft-tissue condition, extensor mechanism integrity, infection severity, and previous surgical history.

**Figure 1:**
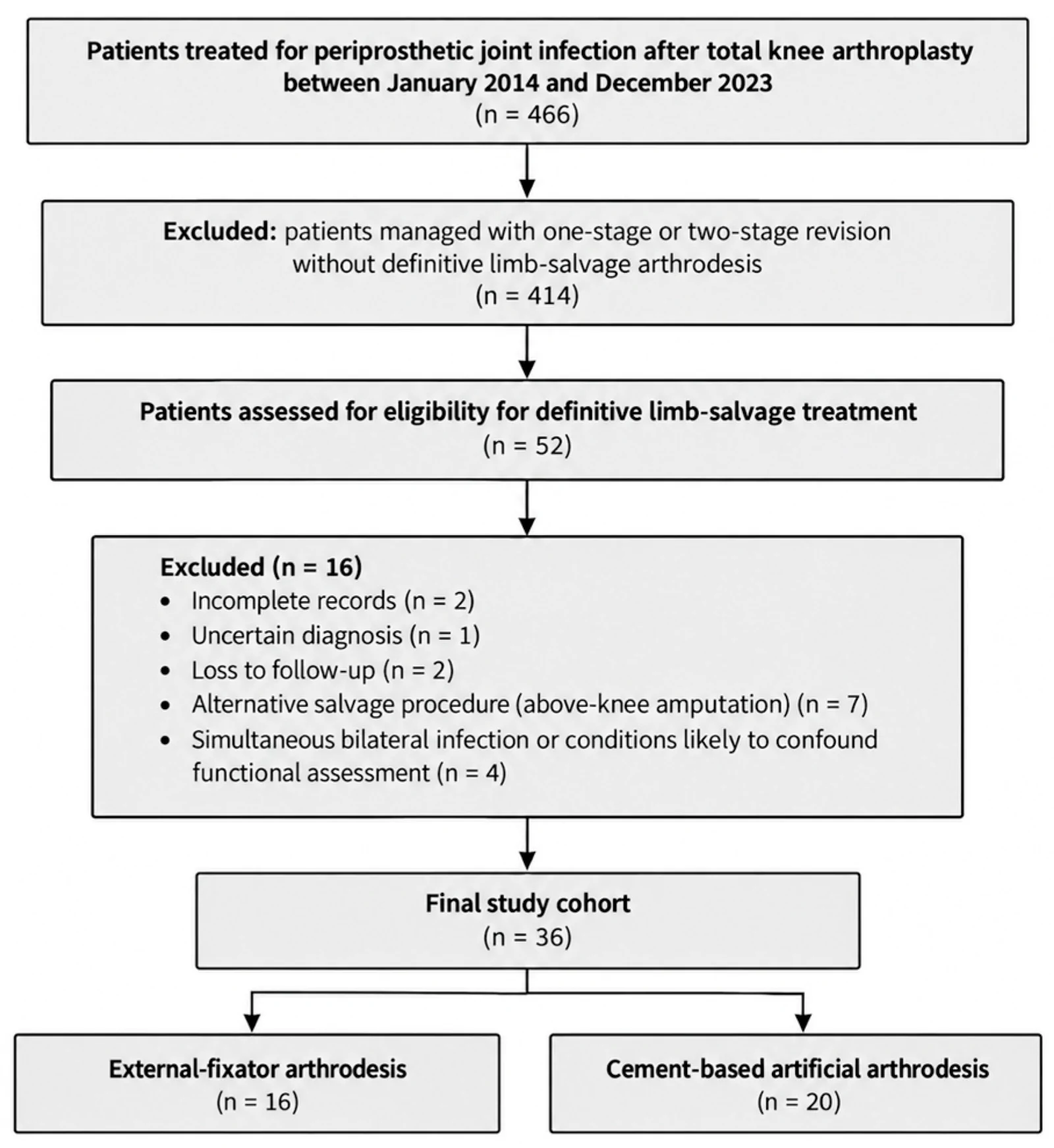
Flow diagram of patient selection.

During the inclusion period, 466 patients were treated for PJI following TKA. Of these, 414 were managed with one-stage or two-stage revision without definitive limb salvage arthrodesis and were therefore not eligible for the present analysis. The remaining 52 patients were assessed for eligibility for definitive limb salvage treatment. Patients with incomplete records (*n* = 2), uncertain diagnosis (*n* = 1), loss to follow-up (*n* = 2), or those who underwent alternative salvage procedures such as above-knee amputation (*n* = 7) were excluded. Simultaneous bilateral infections requiring intervention on both knees and conditions likely to confound functional assessment (*n* = 4) were also excluded. After application of these criteria, 36 patients were included in the final cohort, comprising 16 patients treated with external fixator arthrodesis and 20 treated with cement-based artificial arthrodesis (Figure 1).

### 2.2. Data Collection and Outcome Measures

This exploratory study compared the postoperative functional, structural, and clinical outcomes of the two salvage strategies. Demographic characteristics, surgical history, comorbidities, American Society of Anesthesiologists (ASA) physical status classification, microbiological findings, and preoperative inflammatory markers, including C-reactive protein (CRP) and erythrocyte sedimentation rate (ESR), were obtained from institutional records.

Radiographic and intraoperative findings were reviewed to document bone loss, implant status, soft-tissue condition, and extensor mechanism integrity. Preoperative and postoperative ambulation statuses were categorized as independent ambulation, ambulation requiring walking aids, or wheelchair dependence.

Functional status was assessed using the Short Form-12 (SF-12) and Short Form-36 (SF-36) Health Surveys. Both questionnaires were administered at the final follow-up visit. All patients included in the functional assessment completed both questionnaires, and no PROM data were missing.

The evaluated postoperative outcomes included ambulation status, postoperative limb shortening, complications requiring additional surgical intervention, reinfection requiring surgery, and patient-reported willingness to undergo the same procedure again.

Reinfection was defined as recurrent or persistent PJI after the index salvage procedure requiring additional surgical intervention. Infection-related reoperations were distinguished from procedures performed for mechanical failure, fracture, nonunion, wound complications, or other non-infectious causes.

### 2.3. Surgical Technique

All procedures were performed under general or regional anesthesia with the patient in the supine position. Whenever feasible, the most lateral previous incision was used to minimize further compromise of the soft-tissue envelope. If standard medial parapatellar exposure was insufficient, a rectus snip approach was employed. All retained prosthetic components and previous cement spacers were removed, followed by meticulous circumferential debridement of devitalized soft tissue, fibrous membranes, and necrotic bone. The collateral ligaments were released when intact, and the posterior capsule was thoroughly debrided to eliminate residual infectious foci and facilitate limb alignment.

#### 2.3.1. Cement-Based Artificial Arthrodesis

The femoral and tibial canals were sequentially prepared using hand reamers and curettes to remove residual cement and infected debris. In all patients, a non-locking tibial intramedullary nail was used to provide axial intramedullary support. In this series, tibial nails provided sufficient support, and additional femoral nail fixation was not considered necessary. All cement-based artificial arthrodesis procedures were performed as definitive single-stage salvage procedures rather than as temporary spacer constructs intended for subsequent reimplantation. In this group, “artificial arthrodesis” refers to a mechanically stable femorotibial construct created by the intramedullary nail and cement bridge; osseous fusion was not the intended endpoint.

**Figure 2:**
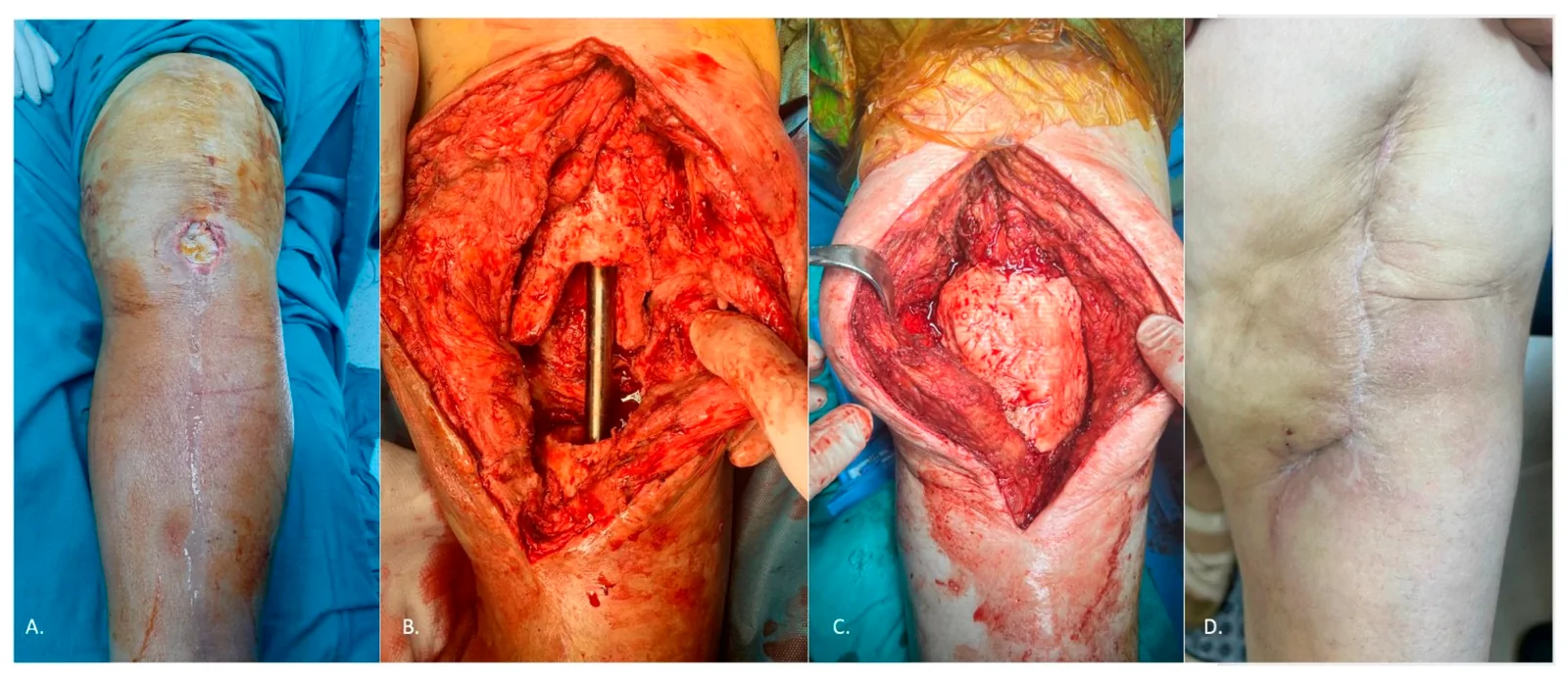
Cement-based artificial knee arthrodesis for recurrent periprosthetic joint infection. (**A**) Draining sinus before surgery. (**B**) Extensive debridement and intramedullary nail insertion. (**C**) Reconstruction of the femorotibial defect with antibiotic-loaded polymethylmethacrylate. (**D**) Healed soft tissues at 20 months.

**Figure 3:**
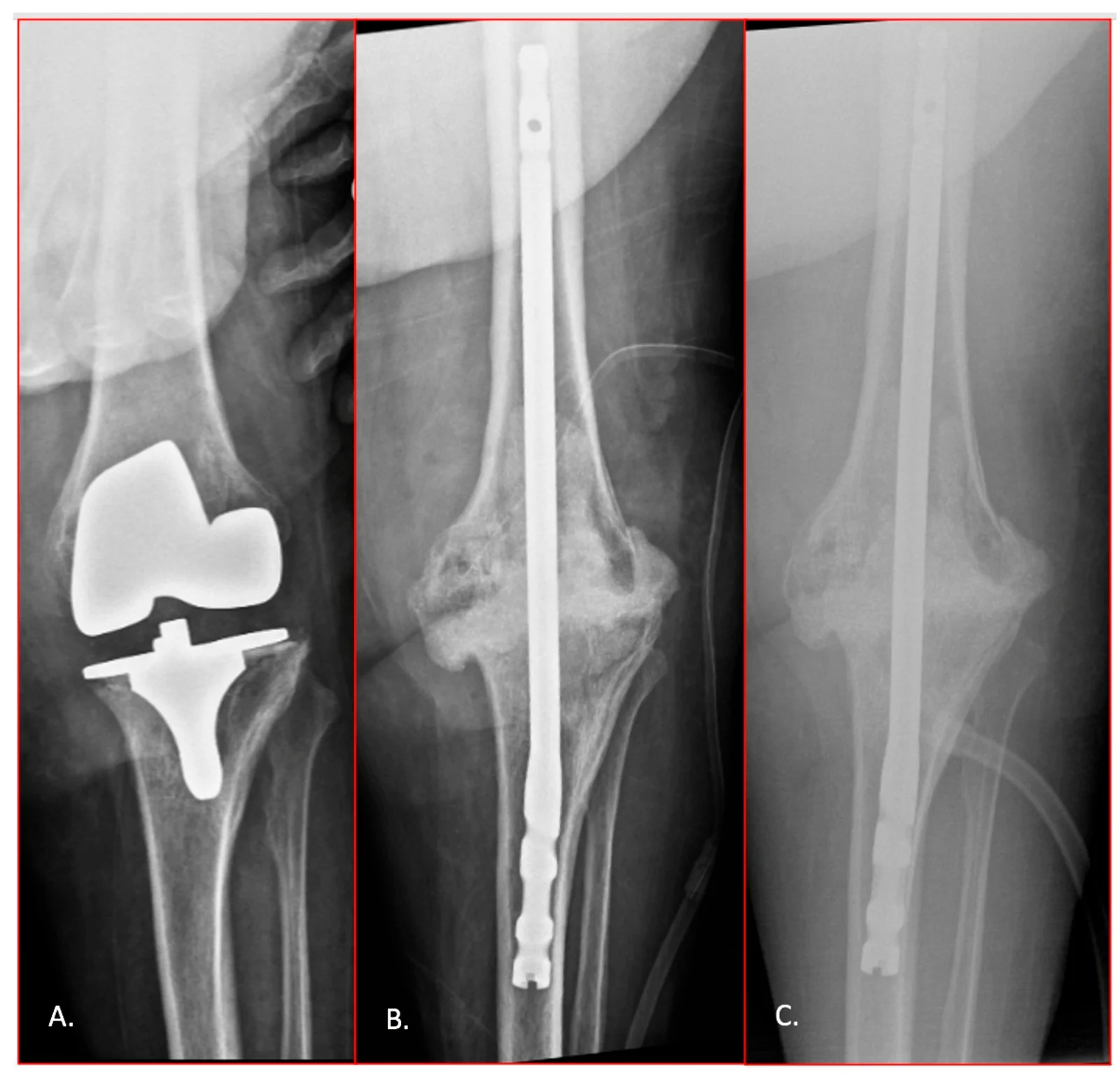
Radiographic evaluation of cement-based artificial arthrodesis. (**A**) Preoperative implant loosening, bone loss, and coronal malalignment. (**B**) Early postoperative radiograph after cement-based arthrodesis with intramedullary nail fixation. (**C**) Maintained alignment and construct stability at one year.

**Figure 4:**
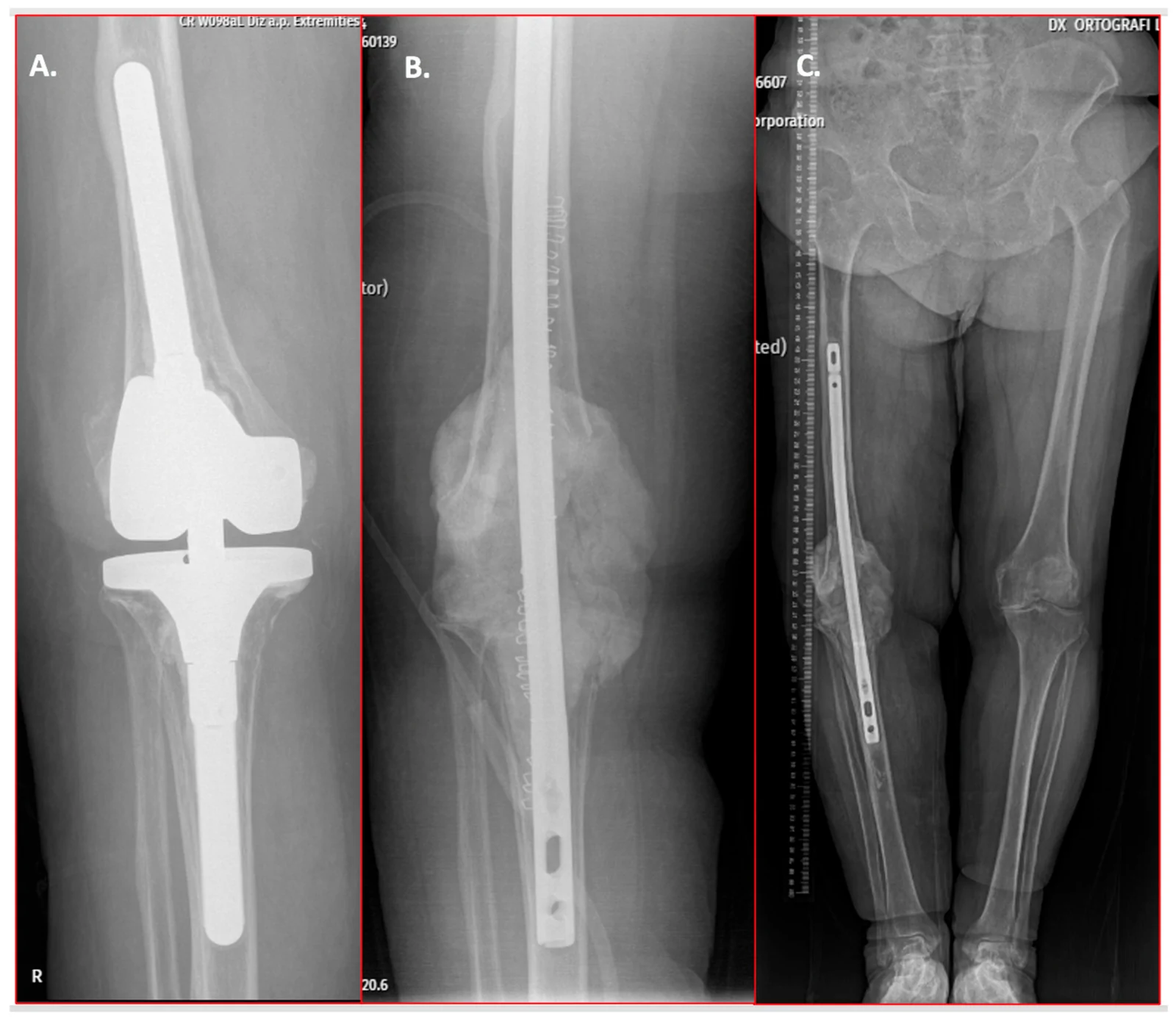
Cement-based artificial arthrodesis after failed two-stage revision. (**A**) Preoperative radiograph showing recurrent infection after failed revision arthroplasty. (**B**) Early postoperative appearance after implant removal and cement-based arthrodesis. (**C**) Maintained construct stability at 48 months despite residual axial malalignment.

Commercially available antibiotic-free polymethylmethacrylate (PMMA) cement was used. Antibiotics were manually added to the cement according to microbiological and antimicrobial susceptibility results obtained from preoperative joint aspiration and previous infection-related surgical procedures, at doses determined preoperatively in consultation with the infectious diseases team. In culture-negative cases, empirical antibiotic selection was determined in consultation with the infectious diseases team based on the clinical and microbiological history. During the index procedure, at least five separate tissue samples were obtained from different anatomical sites for microbiological analysis, and the postoperative systemic antibiotic regimen was subsequently adjusted according to the intraoperative culture and susceptibility results. The antibiotic-loaded cement was molded to fill the femorotibial defect and form a stable cement column between the distal femur and proximal tibia (Figure 2, Figure 3 and Figure 4).

Unlike external fixator arthrodesis, the knee was not positioned in flexion. The construct was created in extension. Intentional limb shortening was accepted when required to obtain tension-free soft-tissue closure. The amount of shortening was not predetermined; rather, it was limited to the minimum necessary to achieve adequate wound closure without excessive tension on the surrounding soft tissues.

After cement polymerization, construct stability and alignment were reassessed. Layered wound closure was performed, and drains were used at the surgeon’s discretion. Patients were mobilized immediately and allowed weight bearing as tolerated. Sutures were removed between postoperative days 14 and 20 if no wound complications occurred.

#### 2.3.2. External Fixator Arthrodesis

After implant removal and extensive debridement, the distal femur and proximal tibia were shaped to obtain broad cancellous contact surfaces. The limb was aligned in slight valgus and approximately 5–10° of flexion, with axial rotation matched to the contralateral side [10]. Limited intentional shortening was accepted when necessary to facilitate stable bone contact and tension-free soft-tissue closure.

A monolateral external fixator was applied spanning the femur and tibia. Six Schanz screws were inserted proximally in the femur and six distally in the tibia under fluoroscopic guidance. Controlled axial compression was applied across the arthrodesis site to obtain stable bone contact.

**Figure 5:**
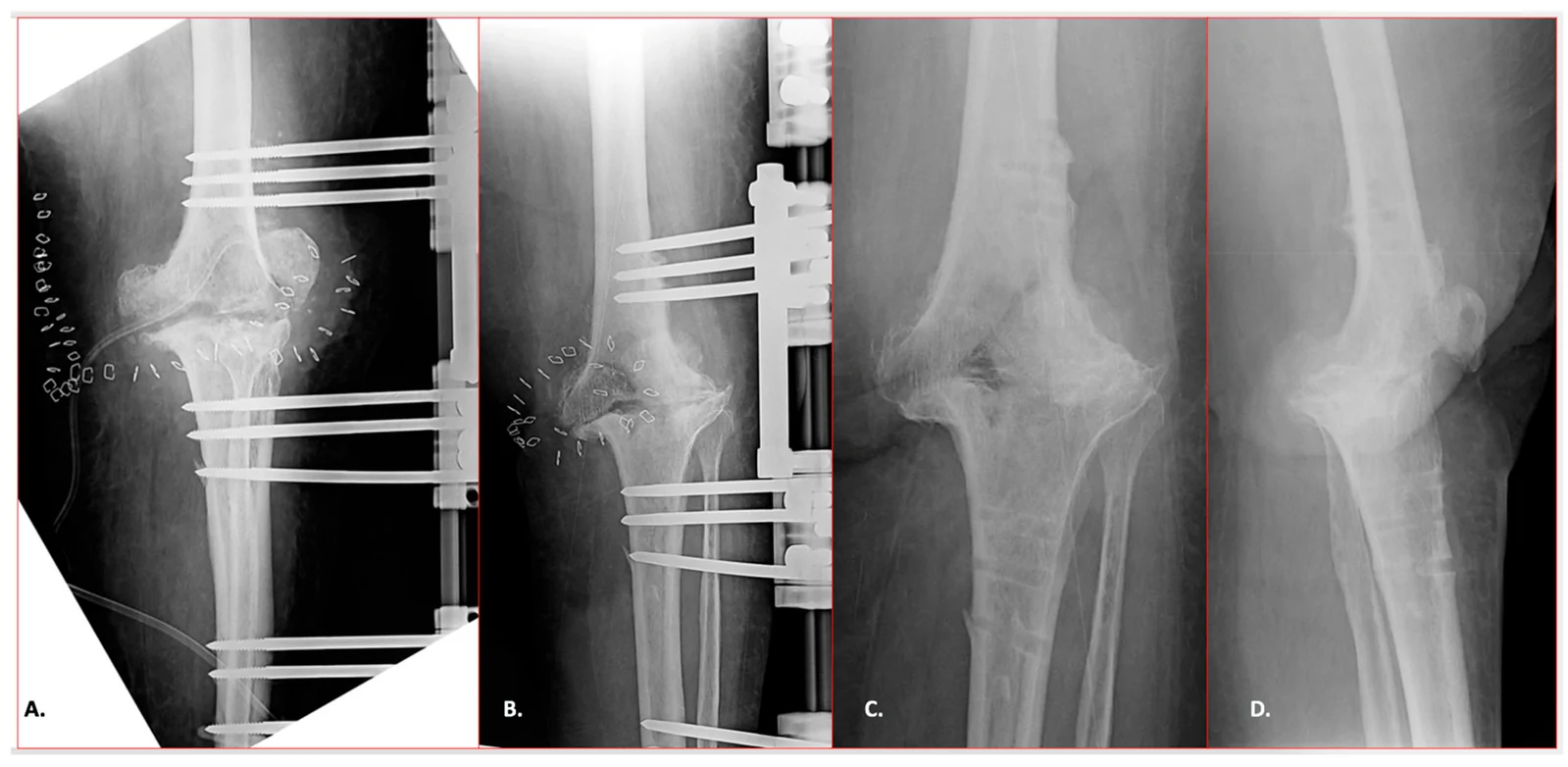
External fixator arthrodesis for persistent periprosthetic joint infection. (**A**,**B**) Early postoperative anteroposterior and lateral radiographs after external fixator arthrodesis and soft-tissue reconstruction. (**C**,**D**) Radiographs at 7 months showing stable alignment and consolidation.

Postoperatively, patients were mobilized with crutches and allowed progressive weight bearing as tolerated. Clinical and radiographic follow-ups were performed regularly. The external fixator was removed once radiographic evidence of osseous union was observed. After fixator removal, a temporary protective orthosis was applied. No patient in the external fixator group underwent subsequent conversion to internal fixation (Figure 5).

### 2.4. Data Analyses

Statistical analyses were performed using Statistical Product and Service Solutions version 28.0 (SPSS, IBM Corporation, Armonk, NY, USA). Continuous variables were assessed for distributional characteristics using skewness and kurtosis values together with visual inspection of histograms and Q-Q plots. Because of the small sample size, these assessments were interpreted collectively rather than relying on a single normality criterion. Normally distributed continuous variables were compared using the independent-samples *t*-test. Welch’s *t*-test was used when the assumption of homogeneity of variance was not met. Non-normally distributed variables were analyzed using the Mann–Whitney U test.

Categorical variables were compared using the chi-square test or Fisher’s exact test when expected cell frequencies were small. Functional outcomes derived from the SF-12 and SF-36 were analyzed according to the distributional characteristics of each domain. For selected principal outcomes, 95% confidence intervals (CIs) were calculated for between-group mean differences using unequal-variance t-based estimates and for absolute risk differences using the Newcombe method. Exact binomial 95% CIs were calculated for group-specific reinfection proportions.

Given the relatively small sample size and non-random treatment allocation, the analyses were considered exploratory and hypothesis-generating. No multivariable adjustment was performed because the limited sample size and substantial baseline imbalance between treatment groups would have resulted in a high risk of model overfitting. Accordingly, between-group comparisons were interpreted as descriptive associations rather than estimates of comparative treatment effectiveness.

Because multiple SF-12 and SF-36 domains were examined, findings from individual domains were also regarded as exploratory, and isolated statistically significant results were interpreted cautiously in view of the potential for type I error. No formal correction for multiple comparisons was applied. All statistical tests were two-tailed, and a *p*-value < 0.05 was considered statistically significant. Non-significant findings were not interpreted as evidence of equivalence between the treatment groups.

## 3. Results

A total of 36 patients met the eligibility criteria and were included in the final analysis. Sixteen patients underwent external fixator arthrodesis and 20 underwent cement-based artificial arthrodesis. The mean age was 67.2 ± 17.2 years in the external fixator group and 73.0 ± 6.8 years in the cement-based artificial arthrodesis group (*p* = 0.18). Mean follow-up durations were 33.4 ± 24.3 months and 37.7 ± 22.6 months, respectively (*p* = 0.58). No statistically significant between-group differences were observed in sex, body mass index, laterality, smoking status, ASA classification, or number of previous surgical procedures (all *p* > 0.05). However, important baseline differences were present between the groups. Preoperative inflammatory markers were higher in the cement-based artificial arthrodesis group, with mean ESR values of 74.2 ± 25.0 versus 50.6 ± 16.5 mm/h (*p* = 0.003) and mean CRP levels of 40.0 ± 19.0 versus 15.4 ± 5.3 mg/L (*p* < 0.001) (Table 1).

Polymicrobial infection was identified in 30.6% of the overall cohort, MRSA in 13.9%, and culture-negative infection in 16.7%. Polymicrobial infection was more frequent in the external fixator group, whereas all culture-negative infections occurred in the cement-based group. The complete microbiological distribution is presented in Table 2.

Preoperative and postoperative ambulation status did not differ significantly between groups (*p* = 0.32 and *p* = 0.09, respectively). Preoperative extensor mechanism insufficiency was significantly more frequent in the external fixator group (81.3% vs. 20%; *p* = 0.001). Postoperative limb shortening was numerically greater in the external fixator group (4.94 ± 3.66 vs. 3.15 ± 0.49 cm), with a mean between-group difference of 1.79 cm (95% CI, −0.17 to 3.75; *p* = 0.07). All patients stated at final follow-up that they would undergo the same procedure again (Table 3).

Postoperative SF-12 and SF-36 scores were broadly similar across most domains. Energy/Fatigue was the only SF-36 domain showing a statistically significant between-group difference, with higher scores in the external fixator group (52.0 ± 7.0 vs. 42.5 ± 12.5; mean difference, 9.50 points; 95% CI, 2.77 to 16.23; *p* = 0.011). Given the multiple domains assessed, this finding was considered exploratory (Table 4).

**Figure 6:**
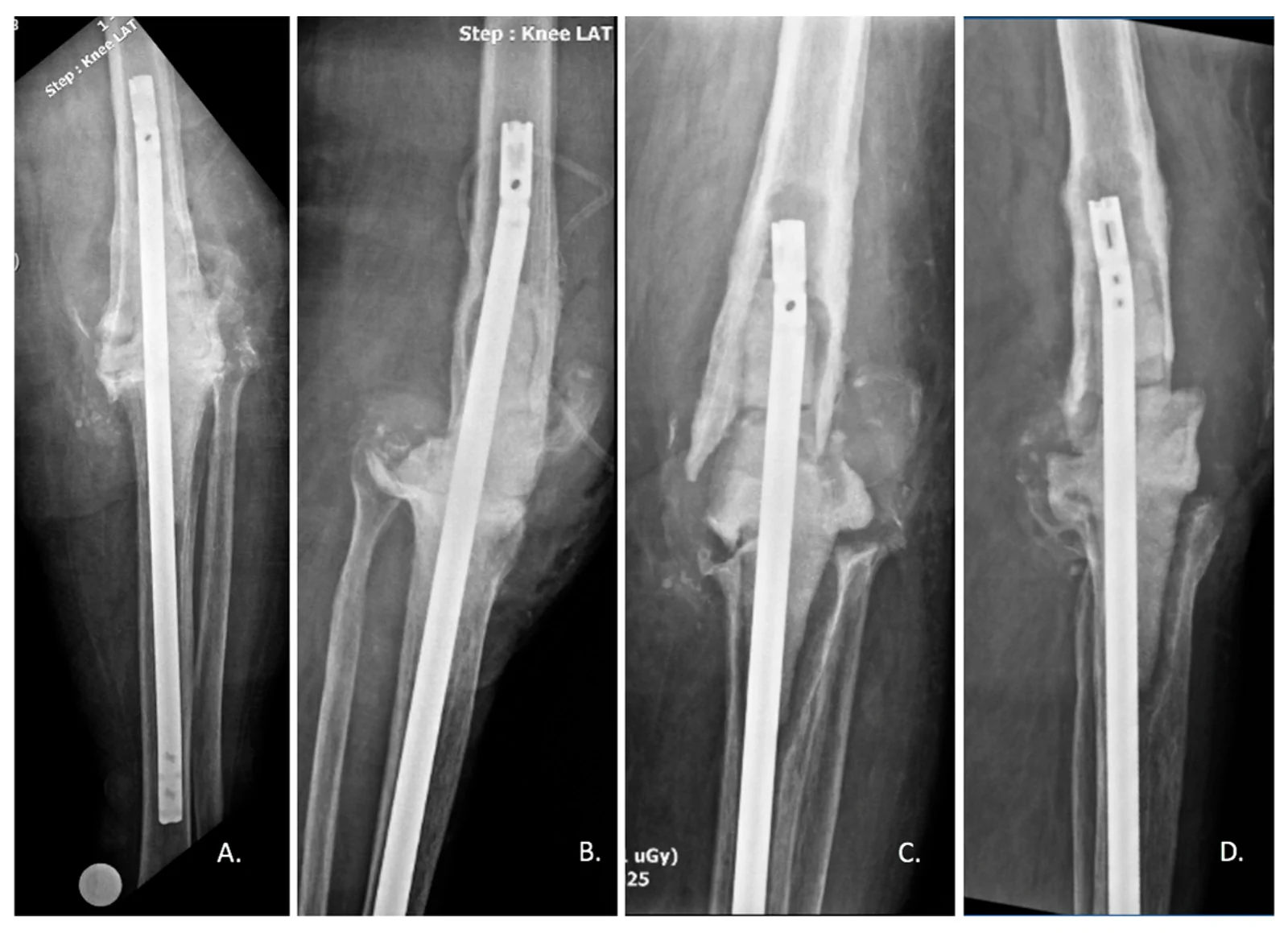
Mechanical failure after cement-based artificial arthrodesis. (**A**,**B**) Early postoperative radiographs showing initial construct stability. (**C**,**D**) Radiographs at 24 months showing implant loosening, radiolucent lines, and progressive osteolysis.

Postoperative complications requiring surgical intervention occurred in 5/16 patients (31.2%) in the external fixator group and 5/20 (25%) in the cement-based artificial arthrodesis group, corresponding to an absolute risk difference of 6.3 percentage points (95% CI, −21.5 to 34.2; *p* = 0.72). Reinfection requiring additional surgery occurred in 0/16 patients (0%; exact 95% CI, 0 to 20.6%) in the external fixator group and 1/20 patients (5.0%; exact 95% CI, 0.1 to 24.9%) in the cement-based artificial arthrodesis group. The absolute risk difference was −5.0 percentage points (95% CI, −23.6 to 14.8). Reinterventions in the external fixator group included secondary lengthening, free-flap reconstruction, surgery for nonunion, and fracture treatment, whereas procedures in the cement-based group were related to wound complications, recurrent infection, postoperative fracture, and aseptic loosening (Figure 6).

## 4. Discussion

The management of persistent or recurrent PJI following TKA remains one of the most challenging scenarios in reconstructive knee surgery. In this exploratory retrospective cohort, external fixator arthrodesis and cement-based artificial arthrodesis showed broadly overlapping postoperative functional profiles, although the two treatment groups differed substantially in several clinically relevant baseline characteristics. In particular, patients treated with cement-based artificial arthrodesis had higher preoperative inflammatory markers, whereas extensor mechanism insufficiency was markedly more frequent in the external fixator group. These imbalances likely reflect differences in treatment selection and introduce important confounding by indication.

Postoperative patient-reported outcome measures did not demonstrate statistically significant between-group differences across most SF-12 and SF-36 domains. The Energy/Fatigue domain was the only SF-36 subscale showing a significant difference, with higher scores in the external fixator group (mean difference, 9.50 points; 95% CI, 2.77 to 16.23). However, given the number of simultaneously evaluated domains and the small sample size, this isolated finding should be regarded as exploratory and interpreted cautiously because of the possibility of type I error. Importantly, preoperative SF-12 and SF-36 scores were unavailable; therefore, the present study cannot quantify within-patient improvement or functional recovery over time and can only describe postoperative status at final follow-up. Sarzaeem et al. reported favorable pain and functional outcomes after artificial fusion using cement-filled intramedullary rods [13], whereas Li et al. demonstrated improvement in pain and knee scores in medically compromised patients treated with permanent static cement spacers [15]. These findings support the potential functional role of definitive cement-based constructs, although direct comparative evidence remains limited.

Ambulation and limb length preservation are important practical considerations after knee arthrodesis. At final follow-up, nearly all patients in both groups remained ambulatory, although the distribution of independent ambulation and walking-aid use differed numerically between groups. Yeung et al. reported preservation of ambulation in most successfully treated patients after knee arthrodesis for infected TKA [18], while a separate two-stage arthrodesis cohort found that 87% of patients who achieved limb salvage remained mobile despite a substantial early reoperation burden [19]. These observations reinforce that preservation of ambulation is a clinically meaningful outcome in this highly selected salvage population.

Postoperative limb shortening was numerically greater in the external fixator group than in the cement-based artificial arthrodesis group (4.94 ± 3.66 cm vs. 3.15 ± 0.49 cm; mean difference, 1.79 cm; 95% CI, −0.17 to 3.75), but the difference was not statistically significant. Nevertheless, the observed difference is clinically plausible and may warrant further investigation in larger cohorts. Conventional bone-to-bone arthrodesis requires adequate osseous contact and may necessitate additional shortening after extensive debridement, particularly in the presence of substantial metaphyseal bone loss. Robinson et al. reported clinically relevant limb length discrepancy after arthrodesis for failed infected TKA [20], while cement-based techniques have been developed to bridge large femorotibial defects without requiring direct bone apposition [13,14]. In the present series, intentional shortening was still required in some cement-based cases to permit tension-free soft-tissue closure, emphasizing that postoperative limb length is influenced not only by the reconstruction method, but also by the condition of the surrounding soft tissues.

The microbiological profile of the present cohort reflects the complexity of salvage treatment after failed revision for PJI. Polymicrobial infection was present in 30.6% of patients and culture-negative infection in 16.7%, with resistant Gram-positive organisms and Gram-negative pathogens also represented. Culture-negative PJI is a recognized diagnostic and therapeutic challenge [21], and microbiologically complex infections are common in salvage populations. In a complex two-stage arthrodesis cohort, polymicrobial and multidrug-resistant infections were frequent [19], while other salvage series have likewise documented heterogeneous pathogen profiles [13,22]. However, the present study was not designed to determine whether particular organisms influenced treatment selection or outcome; therefore, the microbiological findings should remain descriptive rather than causal.

A particularly important baseline difference between groups was the prevalence of extensor mechanism insufficiency, which was present in 81.3% of patients treated with external fixation compared with 20% of those treated with cement-based artificial arthrodesis. Extensor mechanism disruption is a recognized indication for arthrodesis in the setting of failed revision TKA because functional reconstruction may not be feasible. Modular fusion has been specifically described in patients with combined PJI and extensor mechanism disruption [23,24], and external fixation has also been used in the presence of irreparable extensor mechanism deficiency, extensive bone loss, and severe host compromise [11]. However, the marked imbalance observed in the present cohort should not be interpreted as evidence that both techniques perform similarly in patients with extensor mechanism failure. Rather, it likely reflects treatment-selection patterns and represents an important source of confounding by indication.

Complications requiring additional surgery were frequent in both groups, which is consistent with the substantial morbidity reported after salvage arthrodesis for persistent or recurrent PJI. In the external fixator group, reinterventions were related to limb length discrepancy, soft-tissue reconstruction, nonunion, and postoperative fracture. In the cement-based artificial arthrodesis group, reoperations included wound-related procedures, recurrent infection, postoperative fracture, and late aseptic loosening. Reinfection requiring additional surgery occurred in one patient in the cement-based group and in none of the patients in the external fixator group during the observed follow-up period; however, these numbers are too small to support meaningful conclusions regarding comparative infection control. The broader literature similarly demonstrates that complication and reoperation burdens remain substantial regardless of fixation strategy. Early results with modular arthrodesis implants have shown the feasibility of limb salvage in uncontrolled PJI [25], while longer-term intramedullary series have demonstrated durable constructs but persistent risks of reinfection and revision [26,27]. Gathen et al. also described a substantial revision burden after arthrodesis for failed infected knee arthroplasty [22]. A recent systematic review of 23 studies found no statistically significant differences between intramedullary nailing and external fixation in fusion, reinfection, or conversion-to-amputation rates [12]. Collectively, these findings suggest that the pattern of complications may differ by technique, while the overall morbidity of salvage arthrodesis remains considerable.

The principal contribution of the present study lies not in demonstrating superiority of one technique, but in providing comparative real-world data on two infrequently used limb salvage strategies in a highly complex patient population. Cement-based artificial arthrodesis has predominantly been described in small case series using cement-based intramedullary constructs [13,14], as well as reports of retained definitive cement spacers in selected patients [15,16]. In contrast, the broader arthrodesis literature emphasizes both the durability of limb salvage and the substantial reoperation burden associated with these procedures [12,18,19]. Although the present cohort is small, this reflects the relative rarity and highly selected nature of definitive salvage procedures after failed treatment of infected TKA. The study therefore provides hypothesis-generating information regarding patient characteristics, postoperative function, limb shortening, complications, and treatment-selection patterns that may inform future multicenter investigations.

This study has several limitations. First, its retrospective design and non-random treatment allocation introduce substantial selection bias and, more specifically, confounding by indication. The groups differed in clinically relevant baseline characteristics, including inflammatory burden, extensor mechanism integrity, and soft-tissue condition, all of which may have influenced both procedure selection and postoperative outcomes. Because of the small sample size, multivariable adjustment was not performed to avoid overfitting, and residual confounding therefore remains unavoidable. Second, the limited sample size reduces statistical precision and increases the risks of both type I and type II errors. Accordingly, non-significant findings should not be interpreted as evidence of equivalence, while isolated statistically significant findings, particularly across multiple patient-reported outcome domains, should be viewed cautiously. Third, preoperative SF-12 and SF-36 scores were unavailable, preventing assessment of within-patient functional change over time. Fourth, although the minimum follow-up was 12 months, follow-up duration varied between patients, which may influence detection of late mechanical or infectious complications. Fifth, surgical technique was not completely standardized, particularly with respect to debridement extent, cement volume, intentional shortening, and soft-tissue management. Arthrodesis position also differed between techniques, with the cement-based construct created in extension and the external fixator group positioned in slight flexion. Finally, the two-center design and highly selected salvage population limit generalizability to broader revision TKA populations.

Despite these limitations, the present study adds comparative clinical information to a field in which available evidence remains dominated by small retrospective series. Larger multicenter prospective studies with standardized treatment-selection criteria, predefined outcomes, and longer follow-up are required to clarify which patients may benefit most from each salvage approach.

## 5. Conclusions

In selected patients with persistent or recurrent PJI after failed TKA revision, both external fixator arthrodesis and cement-based artificial arthrodesis can be used as limb salvage strategies, although each is associated with substantial morbidity. Cement-based artificial arthrodesis may be particularly useful when severe bone loss or soft-tissue conditions make direct bone-to-bone fusion difficult. In this cohort, postoperative shortening was numerically lower with the cement-based construct, but the difference was not statistically significant, and most patient-reported outcomes did not show statistically significant between-group differences. Because treatment allocation was non-random, baseline characteristics were imbalanced, and the sample was small, so these findings should be regarded as exploratory rather than evidence of equivalence or superiority. Larger prospective multicenter studies are required to define patient selection, long-term durability, and comparative effectiveness.

## Figures and Tables

**Table 1:** Comparison of Demographic and Baseline Characteristics Between Groups.

Variable	External Fixator (*n* = 16)	Cement-Based Artificial Arthrodesis (*n* = 20)	*p*-Value
Age (years)	67.2 ± 17.2	73.0 ± 6.8	0.18
Follow-up (months)	33.4 ± 24.3	37.7 ± 22.6	0.58
Sex, women	12 (75)	13 (65)	0.72
BMI (kg/m^2^)	31.6 ± 2.5	32.7 ± 4.9	0.40
Side, right	10 (62.5)	9 (45)	0.34
Number of previous surgeries	3.4 ± 0.9	3.8 ± 1.5	0.27
ASA classification			0.65
ASA 2	13 (81.3)	15 (75)	
ASA 3	3 (18.8)	4 (20)	
ASA 4	0 (0)	1 (5)	
Smoking	3 (18.8)	3 (15)	1.00
Preoperative ESR (mm/h)	50.6 ± 16.5	74.2 ± 25.0	**0.003**
Preoperative CRP (mg/L)	15.4 ± 5.3	40.0 ± 19.0	**<0.001**

BMI, body mass index; ASA, American Society of Anesthesiologists physical status classification; ESR, erythrocyte sedimentation rate; CRP, C-reactive protein. *p* < 0.05 indicates statistical significance. Bold values indicate statistical significance (*p* < 0.05).

**Table 2:** Distribution of Microorganisms According to Surgical Method.

Microorganism	External Fixator (*n* = 16), *n* (%)	Cement-Based Artificial Arthrodesis (*n* = 20), *n* (%)
Polymicrobial	7 (43.8)	4 (20)
MRSA	4 (25)	1 (5)
MSSA	2 (12.5)	4 (20)
Acinetobacter	2 (12.5)	0 (0)
Pseudomonas	0 (0)	1 (5)
Culture-negative	0 (0)	6 (30)
Serratia	0 (0)	2 (10)
MRKNS	1 (6.2)	0 (0)
Other	0 (0)	2 (10)

MRSA, methicillin-resistant *Staphylococcus aureus*; MSSA, methicillin-sensitive *Staphylococcus aureus*; MRKNS, methicillin-resistant coagulase-negative staphylococci. “Other” denotes organisms not categorized under the prespecified groups.

**Table 3:** Comparison of Functional and Postoperative Outcomes Between Groups.

Variable	External Fixator (*n* = 16)	Cement-Based Artificial Arthrodesis (*n* = 20)	*p*-Value
Preoperative ambulation			0.32
Independent	1 (6.3)	0 (0)	
Crutches	10 (62.5)	10 (50)	
Wheelchair	5 (31.2)	10 (50)	
Postoperative ambulation			0.09
Independent	0 (0)	4 (20)	
Crutches	16 (100)	15 (75)	
Wheelchair	0 (0)	1 (5)	
Would undergo same surgery again	16 (100)	20 (100)	1.00
Preoperative extensor mechanism defect	13 (81.3)	4 (20)	**0.001**
Preoperative soft-tissue defect	3 (18.8)	10 (50)	0.08
Postoperative shortening (cm)	4.94 ± 3.66	3.15 ± 0.49	0.07
Postoperative complications requiring surgical intervention	5 (31.2)	5 (25)	0.72
Reinfection requiring additional surgical intervention	0 (0)	1 (5)	—

Values are *n* (%) unless otherwise indicated. Postoperative shortening is presented as mean ± SD. Reinfection is reported descriptively because only one event occurred. The mean between-group difference in postoperative shortening was 1.79 cm (95% CI, −0.17 to 3.75). For complications requiring surgical intervention, the absolute risk difference was 6.3 percentage points (95% CI, −21.5 to 34.2). Bold values indicate statistical significance (*p* < 0.05).

**Table 4:** Patient-Reported Outcome Measures According to Surgical Technique.

Variable	External Fixator, Mean ± SD	Cement-Based Artificial Arthrodesis, Mean ± SD	*p*-Value
SF-12 Physical	37.44 ± 9.15	36.46 ± 4.56	0.68
SF-12 Mental	44.81 ± 7.58	46.37 ± 7.72	0.55
SF-36 Physical Function	48.75 ± 9.75	46.75 ± 14.53	0.64
SF-36 Physical Role Limits	4.19 ± 16.75	0.00 ± 0.00	0.27
SF-36 Emotional Role Limits	33.31 ± 36.58	30.00 ± 35.77	0.78
SF-36 Emotional Well-being	59.19 ± 7.96	63.40 ± 17.76	0.38
SF-36 Social Function	34.13 ± 14.10	29.05 ± 15.77	0.32
SF-36 Pain	52.13 ± 19.44	65.15 ± 21.94	0.07
SF-36 General Health	43.88 ± 9.54	41.50 ± 11.93	0.52
SF-36 Energy/Fatigue	52.0 ± 7.0	42.5 ± 12.5	**0.011**

SF-12, Short Form-12 Health Survey; SF-36, Short Form-36 Health Survey; SD, standard deviation. Analyses of individual domains were exploratory; no formal adjustment for multiple comparisons was applied. For the Energy/Fatigue domain, the mean between-group difference was 9.50 points (95% CI, 2.77 to 16.23). Bold values indicate statistical significance (*p* < 0.05).

## Data Availability

The data supporting the findings of this study are available from the corresponding author upon reasonable request, subject to applicable ethical and institutional restrictions. The data are not publicly available due to patient confidentiality and privacy considerations.
